# Body mass index and wealth index: positively correlated indicators of health and wealth inequalities in Nairobi slums

**DOI:** 10.1017/gheg.2018.10

**Published:** 2018-06-04

**Authors:** T. N. Haregu, S. F. Mohamed, S. Muthuri, C. Khayeka-Wandabwa, C. Kyobutungi

**Affiliations:** 1 African population and Health Research Center, P.O. Box 10787-00100, Naiorbi, Kenya; 2 School of Pharmaceutical Science and Technology (SPST), Health Sciences Platform, Tianjin University, Tianjin 300072, China

**Keywords:** Body mass index, slums, wealth index

## Abstract

**Introduction:**

Wealth index is a known predictor of body mass index (BMI). Many studies have reported a positive association between BMI and *socioeconomic status* (SES). However, an in-depth investigation of the relationship between BMI and wealth index is lacking for urban slum settings.

**Objective:**

To examine the association between BMI and wealth index in an urban *slum* setting in Nairobi, Kenya.

**Methods:**

A total of 2003 adults between 40 and 60 years of age were included. BMI was derived from direct weight and height measurements. Wealth Index was computed using the standard principal component analysis of household amenities ownership. The relationship between BMI and wealth index was assessed using both linear and logistic regression models.

**Results:**

We found that BMI linearly increased across the five quintiles of wealth index in both men and women, after adjusting for potential confounding factors. The prevalence of obesity increased from 10% in the first wealth quintile to 26.2% in the fifth wealth quintile. The average BMI for women entered the overweight category at the second quintile wealth status, or the third quintile for the total population.

**Conclusion:**

There exists a strong positive relationship between BMI and wealth index in slum settings. Health promotion interventions aimed at reducing obesity may consider using wealth index in priority setting.

## Introduction

Obesity is a rapidly increasing public health challenge in many low- and middle-income countries (LMICs), including those in sub-Saharan Africa (SSA). Current evidence suggests that this increase is mainly due to rapid changes in lifestyles among people living in these countries [1]. Although the degree varies, obesity is increasing in all age categories and gender groups in many LMICs [2–4]. Obesity being a key risk factor for several cardio-metabolic diseases [5], its emergence at this rate and scale points to a rapidly growing threat of a global epidemic of chronic diseases if risk reduction and aversion strategies are not put in place in a timely manner. A critical input to these strategies is current evidence about obesity and its correlates [6].

Wealth index, a summary measure of socioeconomic status (SES), is a well-known predictor of body mass index (BMI) and vice versa. Many studies have reported a strong and positive association between BMI and SES at least in LMICs. While an important global public health concern, higher prevalence of overweight and obesity remains concentrated in higher socioeconomic groups within LMICs [7]. In this regard, some studies have gone beyond the study of associations between BMI and SES, to using SES as a predictor of Obesity [8, 9]. In many communities, an overweight/obese or rounder body frame is desired and perceived as an indicator of economic success rather than an indicator of poorer health [10].

Obesity is a major public health issue not only in high- and middle-income groups but also in slum settings, consistently affecting more women than men in African communities [11]. Despite the existence of studies on the association between SES and BMI in both rural and urban communities in Africa, an in-depth investigation of the relationship between these two indices in urban slum settings is lacking [12], particularly now that more than half of the urban population in SSA resides in slums or slum-like settings. It is, however, important to recognise that a number of studies have focused on the relationship between poverty and underweight in settings slum [13–15].

Evidence about the relationship between wealth index and BMI in slum settings will be useful in developing targeted approaches for the prevention of obesity, and promotion of healthy lifestyles. It will also be useful in informing inclusive development strategies that cater for all. Being in the overweight or obese category of BMI may often not be a deliberate choice, and rather, the result of complex interactions among SES and lifestyles factors. This is specifically true in urban slum settings where choices for healthy lifestyles are limited [16].

In light of these, the aim of this study was to examine the association between BMI and wealth index (as a measure of SES) in an urban slum setting in Nairobi, Kenya. More specifically, this study described the patterns of BMI across different wealth index quintiles by age and gender; determined the strength of association between BMI and wealth index; and identified factors that moderate the relationship between BMI and wealth index. By doing so, the study extends knowledge about the relationship between these two indices to slum settings, which are largely perceived as homogenous clusters of people with very low SES.

## Methods

### Data source

The data source for this study was the cross-sectional study nested within the larger AWI-Gen study (Africa Wits-INDEPTH Partnership for the Genomic Research) study conducted in 2015/16. The partnership included five health and demographic surveillance system (HDSS) field sites of the INDEPTH Network across four countries, Ghana, Burkina Faso, Kenya and South Africa. The aim of the larger study, within the Human Heredity and Health in Africa (H3Africa) *research consortium*, was to identify genetic factors that contribute to body composition, including obesity, which together with environmental factors, contribute to susceptibility for cardio-metabolic diseases [17–19]. This study draws on the data from the population-based study at Nairobi sites collected from 2003 adults between the ages of 40–60 in two urban slums of Nairobi: Korogocho and Viwandani.

### Measurements

Among other variables in the larger AWI-gen study, data on weight, height, and basic socio-economic variables were collected from *2003* adults between the ages of 40–60 years in two urban slums of Nairobi in 2015 and 2016. Weight and height, along with other anthropometric measurements, were measured by experienced and trained field workers. BMI was computed as weight in kilograms divided by height in meters squared.

### Categorization of BMI and wealth index

Cut-off points for BMI were defined based on World Health Organization (WHO) recommendations. Obesity was defined by BMI of greater than or equal to 30 kg/m^2^ and overweight was defined by BMI greater than or equal to 25 kg/m^2^ [20]. In this study, BMI was treated both as a categorical and continuous variable for the different models.

The wealth index was computed using principal component analysis, using key variables that assessed ownership of among 34 household infrastructure and amenities including number of rooms in the household, availability of water and sanitation facilities, and ownership of different household items. The resulting wealth index variable was categorized into five quintiles: Very poor, poor, medium, rich and very rich. This is a standard method used in Demographic and Health Surveys (DHS), and based on internationally agreed values [21].

### Data analysis

Data were analysed using Stata 13.0. The prevalence of overweight and obesity across the different wealth quintiles were described using proportions. The association between categories of BMI and Wealth index was examined using a logistic regression model. In addition, the association between log-transformed BMI and quintiles of wealth index was assessed using multiple linear regression models. Independent variables included in the models were age (continuous), gender, educational status, employment status, ethnicity, family size, morbidity and behavioural risk factors. Before the linear regression, linearity assumption was checked using scatter plots. BMI data were log-transformed due to their skewed distribution. Evidence of effect modification was also examined. *p* values smaller than 0.05 were considered to be statistically significant.

## Results

### Characteristics of the study population

A total of 2003 adults between the age of 40 and 60 years were included in this study. Table 1 presents the distribution of key predictor and outcome variables by gender. The majority, 1081 (54%) were women. About two-thirds (62.6%) of the study participants were older than 50 years. The highest level of education for more than half the study population was a primary-level education. Only a third of the study participants had attained a secondary level of education. Nearly half (47.2%) were self-employed and 31.1% were engaged in informal employment.

**Table 1.** Distribution of key predictor and outcome variables by gender

Basic variables	Women		Men		Total		χ^2^ tests
	Number	Per cent	Number	Per cent	Number	Per cent	
Age groups							
40–45	397	36.73	327	35.47	724	36.15	χ^2^ = 10.90 *p* = 0.012
46–50	308	28.49	222	24.08	530	26.46	
51–55	230	21.28	206	22.34	436	21.77	
56–60	146	13.51	167	18.11	313	15.63	
Total	1081	100	922	100	2003	100	
Ethnicity							
Kikuyu	480	44.4	245	26.57	725	36.2	χ^2^ = 77.98 *p* < 0.001
Kamba	196	18.13	197	21.37	393	19.62	
Luo	159	14.71	215	23.32	374	18.67	
Luhya	143	13.23	179	19.41	322	16.08	
Others	103	9.53	86	9.33	189	9.44	
Total	1081	100	922	100	2003	100	
Household size							
1	123	11.39	299	32.46	422	21.09	χ^2^ = 149.47 *p* < 0.001
2	160	14.81	128	13.9	288	14.39	
3	183	16.94	113	12.27	296	14.79	
4	182	16.85	112	12.16	294	14.69	
5	143	13.24	107	11.62	250	12.49	
6	134	12.41	86	9.34	220	10.99	
7 or more	155	14.35	76	8.25	231	11.55	
Total	1080	100	921	100	2001	100	
Education							
No formal education	118	10.92	36	3.9	154	7.69	χ^2^ = 104.29 *p* < 0.001
Primary	682	63.09	469	50.87	1151	57.46	
Secondary	277	25.62	395	42.84	672	33.55	
Tertiary	4	0.37	22	2.39	26	1.3	
Total	1081	100	922	100	2003	100	
Occupation							
Self-employed	632	58.46	314	34.17	946	47.3	χ^2^ = 242.48 *p* < 0.001
Full-time (formal)	50	4.63	217	23.61	267	13.35	
Part-time	19	1.76	26	2.83	45	2.25	
Informal	287	26.55	336	36.56	623	31.15	
Unemployed	93	8.6	26	2.83	119	5.95	
Total	1081	100	919	100	2000	100	
BMI categories							
Underweight	41	3.79	108	11.71	149	7.44	χ^2^ = 338.14 *p* < 0.001
Normal weight	360	33.3	583	63.23	943	47.08	
Overweight	333	30.8	180	19.52	513	25.61	
Obese	347	32.1	51	5.53	398	19.87	
Total	1081	100	922	100	2003	100	
Wealth index							
First quintile	145	13.41	96	10.41	241	12.03	χ^2^ = 34.90 *p* < 0.001
Second quintile	275	25.44	176	19.09	451	22.52	
Third quintile	245	22.66	219	23.75	464	23.17	
Fourth quintile	226	20.91	179	19.41	405	20.22	
Fifth quintile	190	17.58	252	27.33	442	22.07	
**Total**	**1081**	**100**	**922**	**100**	**2003**	**100**	

*Note*: First quintile is lowest and fifth is the richest.

### Prevalence of overweight and obesity

The overall prevalence of obesity in the study population was 20.0%. The prevalence of overweight was 25.5%. The prevalence of obesity was significantly higher among women (32.2 *v.* 5.6%). The prevalence of obesity among those 40–50 years of age was 19.1% while among those aged 51–60 was 21.4%. In the study population, the prevalence of obesity increased with age except among the 46–50 years age group.

### Wealth index in the study population

The distribution of participants in the five wealth index categories shows that 241 (12.3%) very poor, 451 (22.5%) poor, 464 (23.2%) medium, 405 (20.2%) rich and 442 (22.2%) very rich. These categories are relative categories of wealth index within the slum population.

### Patterns of BMI by wealth quintiles

Analysis of mean BMI by wealth quintiles, as displayed in online Supplementary Fig. S1, showed that mean BMI increases as wealth index increases from lower to higher quintiles. The increase is higher in the transition within lower quintiles (from first to second and second to third) than in the last ones (from fourth to fifth). The overall average BMI for the study population (25.4 kg/m^2^) was in the overweight category. The average BMI entered into the overweight category at the third quintile.

**Fig. 1.**
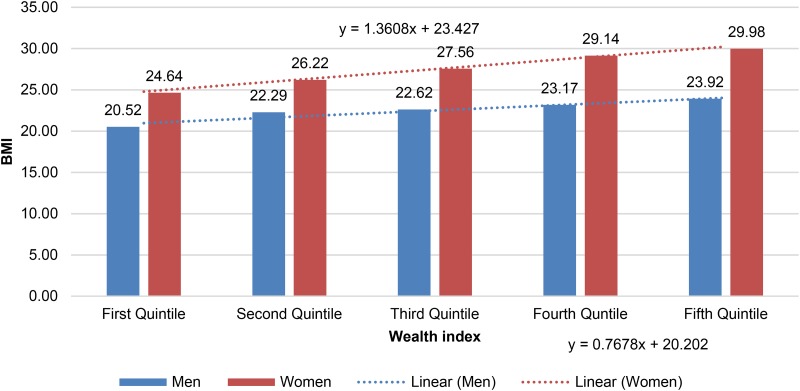
Mean BMI by wealth index and by gender.

The pattern of increasing mean BMI across wealth quintiles was linear for both men and women. As shown in Fig. 1, the linearly increasing mean BMI was at a higher level for women than men. On average mean BMI increases by 1.36 units and 0.77 units for each increase in wealth quintile for women and men, respectively. Women entered the overweight category at the second quintile.

**Fig. 2.**
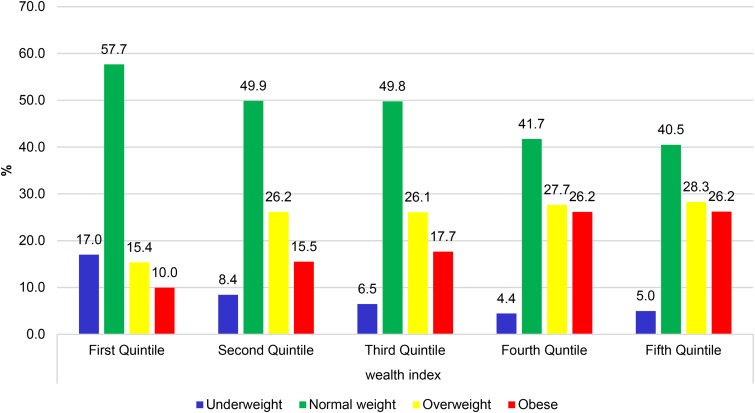
Patterns of BMI Categories by wealth index.

Similarly, analysis of patterns of BMI categories (underweight, normal weight, overweight and obesity) has shown an increasing prevalence of overweight and obesity across the wealth quintiles. As shown in Fig. 2, the prevalence of obesity was 10% in the first quintile as compared to 26.2% in the fifth quintile. Prevalence of overweight had a similar pattern. However, the proportion of people within normal and underweight categories decreased as wealth index increased.

### Multivariate analysis of the relationship between BMI and wealth index

Multivariate analysis conducted separately for women and men indicated that BMI and wealth index were strongly associated. As indicated in Table 2, after controlling for the effect of all potential confounders, the association between high BMI and wealth index persistently increased across the wealth quintiles. While men in the fifth quintile had about four times higher odds of having a high BMI, women in the same quintile had more than five times higher odds of having a high BMI as compared to those in the first quintile.

**Table 2.** High BMI (>25) and Wealth Index: Summary of multiple logistic regression outputs (n = 2003)

	Men AOR (95% CI)	Women AOR (95% CI)	Total AOR (95% CI)
First quintile (REF)	1.0	1.0	1.0
Second quintile	2.7 (1.1–6.6) (*p* = 0.028)	1.9 (1.2–3.0) (*p* = 0.005)	1.9 (1.2–3.0) (*p* = 0.005)
Third quintile	2.5 (1.1–6.2) (*p* = 0.036)	2.6 (1.6–4.2) (*p* < 0.001)	2.6 (1.6–4.1) (*p* < 0.001)
Fourth quintile	3.3 (1.3–8.1) (*p* = 009)	4.5 (2.7–7.4) (*p* < 0.001)	4.4 (2.6–7.4) (*p* < 0.001)
Fifth quintile	4.1 (1.6–10.1) (*p* = 0.002)	5.4 (3.1–9.6) (*p* < 0.001)	5.4 (3.1–9.5) (*p* < 0.001)

Models are adjusted for age ethnicity, marital status, _ education, household size, employment status, _morbidity (diabetes, TB, HIV), behavioural risk factors (smoking, _ alcohol, physical activity and diet) and number of pregnancies, menopause status.

*Note*: First quintile is lowest and fifth is the richest.

Similarly, the association between log-transformed BMI and wealth quintiles after all the potential confounders were controlled for in the multiple linear regression models showed a strong positive association with associations being statistically significant except for those in the second quintile. As shown in Table 3 below, the strength of association between BMI and wealth index consistently increased across the wealth quintiles. As is the case with the previous analysis outputs, the association between BMI and wealth index was stronger for women than men. However, at lower wealth quintiles, the strength of association between BMI and wealth index was stronger for men than women.

**Table 3.** BMI (log) and Wealth Index: Summary of multiple linear regression outputs (n = 2003)

First quintile (REF)	Men		Women		Total	
	Coefficient	*P*	Coefficient	*p*	Coefficient	*p*
Second quintile	0.063 (0.023–0.103)	<0.001	0.040 (−0.001–0.082)	0.054	0.040 (−0.001–0.082)	0.054
Third quintile	0.074 (0.036–0.113)	<0.001	0.078 (0.036–0.121)	<0.001	0.078 (0.036–0.121)	<0.001
Fourth quintile	0.091 (0.050–0.131)	<0.001	0.123 (0.079–0.167)	<0.001	0.123 (0.079–0.167)	<0.001
Fifth quintile	0.104 (0.063–0.145)	<0.001	0.143 (0.096–0.190)	<0.001	0.143 (0.096–0.190)	<0.001

Models are adjusted for age ethnicity, marital status, _ education, household size, employment status, _morbidity (diabetes, TB, HIV), behavioural risk factors (smoking, _ alcohol, physical activity and diet) and number of pregnancies, menopause status.

*Note*: First quintile is lowest and fifth is the richest.

## Discussion

### Summary of the findings

The findings of this study revealed a strong positive association between BMI and wealth index confirming a strong positive relationship in the two indices in urban slum settings. The average BMI of women tends to enter the high BMI category (overweight) at the second quintile while that of the overall study population at the third quintile. The strength of association between these two indices consistently increased with wealth quintiles, even after accounting for the effects of other potential confounding factors. The association was stronger in women, who generally had higher average BMI than men.

### Interpretation in the context of other studies

A recent systematic review found a positive association between obesity and SES in low-income countries for both men and women. This implies that the more affluent and/or those with higher educational attainment tend to be more likely to be overweight and obese [9]. Yet, another systematic review of the relationship between obesity and SES at an ecological level indicated a shift towards obesity to include women of low SES, apparently occurring at an earlier stage of economic development than it did for men [8] which supports what is observed in the current study. Even earlier systematic reviews found that, in developing societies, a strong direct relationship exists between SES and obesity among men, women and children [22]. However, in high-income countries, obesity is inversely associated with SES, women at lower SES being at highest risk for obesity [23].

Studies have reported that belonging to a lower SES group confers protection against obesity in low-income economies [24]. Increasingly positive associations between obesity and SES, for both men and women, as one moved from countries with high levels of socioeconomic development to countries with medium and low levels of development has been reported. These results underscore the view that obesity is a social phenomenon, for which appropriate action ought to target both economic and sociocultural factors [25].

Though it is not fully understood in low-income settings, the relationship between SES and BMI could be mediated by dietary behaviour and physical activity [22, 26]. Those with better SES may have better access to high-calorie diet and at the same time could have higher chances of engaging in paid work that involves more sitting. For the lowest SES groups, the opposite could be true.

The findings of this study support the positive association between BMI and wealth quintiles in general, and between obesity and wealth quintiles in particular, in a low-income setting. Our findings revealed an increasing pattern of overweight and obesity in urban slums where most of the population have lower education status and are highly involved in informal employment. In light of the transitions to a higher prevalence of obesity in low-income groups, especially among women, this study extends our knowledge of understanding of the relationship between body composition and SES in urban slum settings. However, further research is needed to explain additional factors that contribute to the differences in BMI across wealth quintiles.

### Implications for policy, research and practice

People in the higher wealth index categories, especially women, have a higher likelihood of being overweight or obese. This could mean that as SES of people living in slums improves; their chance of being overweight and obese will increase, or that socioeconomic development in urban slums would be associated with an increase in BMI. In this regard, interventions that aim to improve the socio-economic status of individuals in our study setting for urban slums need to integrate health promotion programs targeted at prevention of obesity through improvement of lifestyles.

The findings of this study support the notion that wealth index (**SES**) could potentially be used as a predictor of overweight and obesity in slum settings and the potential impact on cardio-metabolic disease risk. Though this would need further exploration, this predictive power will have particular importance in screening and rapid assessments of health and SES in these study populations.

Future policies targeting slum populations need to prioritize interventions for overweight and obesity among higher wealth index groups, and underweight for those in the lower wealth quintiles. The existence of a strong and positive association between BMI and wealth index is a good signal for using SES in a segmented approach for health promotion interventions in urban slum settings. For instance, increased wealth should lead to behaviour that seeks healthier food options and increased exercise.

The linearly increasing prevalence of obesity across wealth quintiles, and the linearly increasing odds of higher BMI across wealth quintiles mean the intensity of obesity reduction interventions should increase across wealth quintiles. However, further studies are required to determine how and by how much the intensity or dose of these interventions should vary across wealth quintiles.

### Limitations of the study

There are some limitations in this study. First, the study was cross-sectional in nature and the study population was limited to those between 40 and 60 years old and thus may not represent the general population in slums, yet it still provides important information about this age category. Second, the measurement of wealth index took into account the ownership and availability of household amenities and not the quality of those items. Third, BMI, which was used in this study is a measure of general obesity, which limits concerns related to centrally obesity. Finally, we didn't adjust for potential clustering effect at the household level. The findings of this study should, therefore, be interpreted in the context of these limitations.

## Conclusion and recommendations

Based on the findings of this study, it is evident that there exists a strong positive relationship between BMI and wealth index in slum settings, even after accounting for anticipated confounders. The strength of association between BMI and wealth index was higher among women across all wealth quintiles suggesting that, in each wealth quintile, women carry a higher burden of overweight/obesity and its consequences. Given the strength of association between BMI and wealth index in urban slum settings, wealth index could be used as a good predictor of BMI. It is therefore important that health promotion interventions aimed at reducing obesity consider using wealth index distributions in the population as a factor that affects health status including obesity prevalence in low-income settings.

## Supplementary material

For supplementary material accompanying this paper visit https://doi.org/10.1017/gheg.2018.10. click here to view supplementary material
