# Supplementary material for: Body mass index and wealth index: positively correlated indicators of health and wealth inequalities in Nairobi slums
Source: Glob Health Epidemiol Genom. 2018 Jun 4;3:e11. doi: 10.1017/gheg.2018.10 (PMC6152486; doi:10.1017/gheg.2018.10)
Supplement: Supplementary file 1 [file S2054420018000106sup001.docx]

Supplement

1. List of variables included in the principle components analysis

| 1. Dependency ratio 2. How many rooms are there in the house and outside structures used by household members? 3. How many rooms are used for sleeping in? 4. Which of the following items, in working order, do you have in your household at the present time? |
| --- |

Table S1: List of variables used in computing SES

| Electricity | Solar energy | Alternative power source | Television | Radio |
| --- | --- | --- | --- | --- |
| Motor vehicle | Motorcycle | Bicycle | Refrigerator | Washing machine |
| Sewing machine | Mobile phone | Microwave | DVD player | Satellite TV or DSTV |
| Computer or laptop | Internet by mobile phone | Electric iron | Fan | Electric or gas stove |
| Kerosone stove | Electric plate | Torch | Gas lamp | Kerosene lamp with glass |
| Grinding mill | Table | Sofa set | Wall clock | Bed |
| Mattress | Blankets | Cattle | Other livestock* | Poultry** |

* Other livestock includes donkeys, goats, sheep and pigs

** Poultry includes ducks, chickens, geese and other fowl

1. Table S2: Obesity by age categories

| **Age Categories** |  | **Underweight** | **Normal weight** | **Overweight** | **Obese** | **Total** |
| --- | --- | --- | --- | --- | --- | --- |
| 40-45 | # | 48 | 364 | 170 | 142 | 724 |
|  | % | 6.63 | 50.28 | 23.48 | 19.61 | 100 |
| 46-50 | # | 39 | 241 | 153 | 97 | 530 |
|  | % | 7.36 | 45.47 | 28.87 | 18.3 | 100 |
| 51-55 | # | 36 | 205 | 104 | 91 | 436 |
|  | % | 8.26 | 47.02 | 23.85 | 20.87 | 100 |
| 56-60 | # | 26 | 133 | 86 | 68 | 313 |
|  | % | 8.31 | 42.49 | 27.48 | 21.73 | 100 |
| Total | # | 149 | 943 | 513 | 398 | 2,003 |
|  | % | 7.44 | 47.08 | 25.61 | 19.87 | 100 |

1. Figure S1: Patterns of mean BMI for the five Wealth quintiles
